# Genetic Characterization of Conserved Charged Residues in the Bacterial Flagellar Type III Export Protein FlhA

**DOI:** 10.1371/journal.pone.0022417

**Published:** 2011-07-19

**Authors:** Noritaka Hara, Keiichi Namba, Tohru Minamino

**Affiliations:** 1 Graduate School of Frontier Biosciences, Osaka University, Suita, Osaka, Japan; 2 Precursory Research for Embryonic Science and Technology, Japan Science and Technology Agency, Kawaguchi, Saitama, Japan; University of Cambridge, United Kingdom

## Abstract

For assembly of the bacterial flagellum, most of flagellar proteins are transported to the distal end of the flagellum by the flagellar type III protein export apparatus powered by proton motive force (PMF) across the cytoplasmic membrane. FlhA is an integral membrane protein of the export apparatus and is involved in an early stage of the export process along with three soluble proteins, FliH, FliI, and FliJ, but the energy coupling mechanism remains unknown. Here, we carried out site-directed mutagenesis of eight, highly conserved charged residues in putative juxta- and trans-membrane helices of FlhA. Only Asp-208 was an essential acidic residue. Most of the FlhA substitutions were tolerated, but resulted in loss-of-function in the ΔfliH-fliI mutant background, even with the second-site *flhB*(P28T) mutation that increases the probability of flagellar protein export in the absence of FliH and FliI. The addition of FliH and FliI allowed the D45A, R85A, R94K and R270A mutant proteins to work even in the presence of the *flhB*(P28T) mutation. Suppressor analysis of a *flhA*(K203W) mutation showed an interaction between FlhA and FliR. Taken all together, we suggest that Asp-208 is directly involved in PMF-driven protein export and that the cooperative interactions of FlhA with FlhB, FliH, FliI, and FliR drive the translocation of export substrate.

## Introduction

The flagellum of *Salmonella enterica* is a supermolecular motor powered by an electrochemical potential difference of protons (PMF) across the cytoplasmic membrane. The flagellum consists of at least three parts: the basal body, the hook, and the filament. Flagellar assembly begins with the basal body, followed by the hook and finally the filament. Almost all the substructures of the flagellum lie beyond the cytoplasmic membrane. Most of flagellar proteins are transported to the distal end of the growing flagellum by the flagellar type III protein export apparatus [1]–[4]. The components of the export apparatus are highly homologous not only to those of the type III secretion system of pathogenic bacteria, which directly injects virulence effectors into eukaryotic host cell [5] but also to those of FOF1-ATP synthase, which consists of a water soluble F1 part, which is a ring complex having three catalytic sites for ATP synthesis/hydrolysis, and a membrane-integrated FO part, which mediates proton translocation [6]–[8].

The flagellar type III protein export apparatus consists of three soluble proteins (FliH, FliI, FliJ) and six integral membrane proteins (FlhA, FlhB, FliO, FliP, FliQ, FliR) (Figure S1) [9], [10]. The export apparatus is believed to be located in the putative central pore of the basal body MS ring [11]–[13]. FliI is an ATPase [14] and forms a complex with FliH and FliJ [7], [10], [15], [16]. FliI and FliJ bind to the FlgN-FlgK and FliT-FliD chaperone-substrate complexes [17]–[19]. The FliH-FliI-FliJ delivers export substrates to the export gate complex made up of the six integral membrane proteins [20], [21]. A specific interaction of the FliH_X_-FliI_6_-FliJ ring complex with the docking platform formed by the cytoplasmic domains of FlhA and FlhB induces the initial entry of the substrates into the gate [7], [22], [23]. The export gate complex utilizes PMF across the cytoplasmic membrane as the energy source for the translocation of the substrates [22], [24]. It has been shown that a *fliO* null mutant displays a weakly motile phenotype, suggesting that FliO is not directly involved in flagellar protein export [25]. Interestingly, a homologue of FliO is apparently absent in some other type III secretion systems [25].

**Figure 1:**
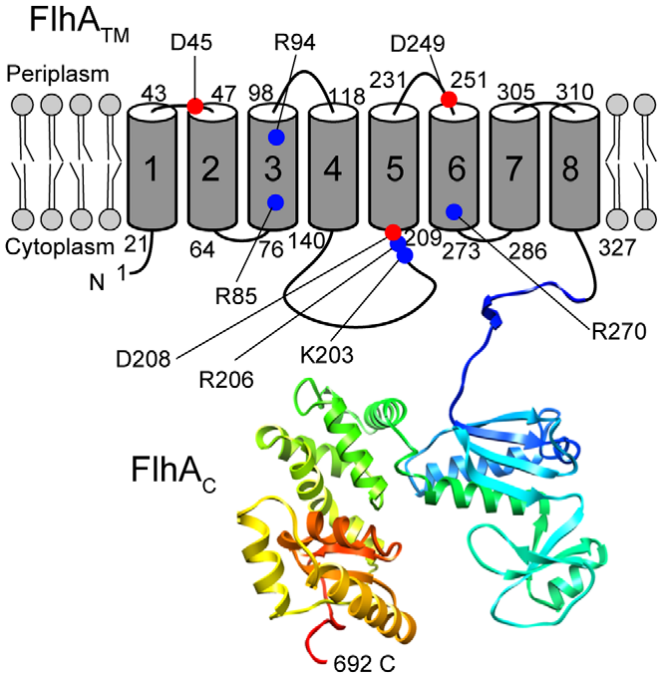
Topology of FlhA. Cartoon showing the domain organization of Salmonella FlhA and the location of the highly conserved charged residues that have been genetically analyzed in this work. FlhA consists of an N-terminal transmembrane domain (FlhATM, TM1–TM8) and a C-terminal cytoplasmic domain (FlhAC) whose atomic structure was solved by X-ray crystallography [41]. FlhAC is involved in the early process of flagellar protein export along with FliH, FliI, FliJ, and the C-terminal cytoplasmic domain of FlhB. Closed circles in red and blue indicate invariant acidic and basic residues, respectively, which were identified by multiple sequence alignment of FlhA homologs (Figure S1).

FlhA is composed of an N-terminal integral membrane domain with eight predicted transmembrane (TM) helices (FlhATM, residues 1–327, 34.5 kDa) and a C-terminal cytoplasmic domain (FlhAC, residues 328–692, 40.5 kDa) (Figure 1) [26]. A well-conserved hydrophilic cytoplasmic loop between TM-4 and TM-5 is indispensable for FlhA function, but little is known about its role in flagellar protein export [27]. FlhAC interacts with FliH, FliI, FliJ, the C-terminal cytoplasmic domain of FlhB (FlhBC), and the FliS-FliC and FliT-FliD chaperone-substrate complexes and initiates the translocation of the substrates [10], [23], [28], [29]. FlhAC consists of four subdomains (D1, D2, D3, and D4) and a linker connecting FlhAC to FlhATM (Figure 1) [29]–[31]. The linker is involved in an interaction with FliJ [29]. The D2 subdomain is responsible for an interaction with the FliT-FliD and FliS-FliC complexes [29]. The D4 subdomain is dispensable for its function but is involved in the substrate specificity switching of the export apparatus [32]. Although FlhATM is required for the association of FlhA with the MS ring [12], it remains unknown whether it is directly involved in the PMF-driven protein export process.

The flagellar type III protein export apparatus shows many similarities with FOF1-ATP synthase [3], [6]–[8]. An inward-directed proton translocation through the FO part drives the rotation of the FO-c-ring as a rotor, and the γ and ε subunits act as a drive shaft to cause conformational changes in the F1 part that result in ATP synthesis. ATP hydrolysis by F1 drives the reverse rotation of the rotor, resulting in an outward-directed proton pumping. Two highly conserved charged residues, Arg-210 in the a subunit and Asp-61 in the c subunit of the E. coli FOF1-ATP synthase are critical for proton translocation [33]. Since FlhATM is the main component of the PMF-driven flagellar protein export gate, there is the possibility that the highly conserved charged residues of FlhATM may be involved in the energy transduction mechanism.

In order to clarify the role of FlhATM in PMF-driven flagellar protein export, we performed genetic analyses of eight highly conserved charged residues in the putative juxta- and trans-membrane helices of FlhA. We show that only a negatively charged residue at position 208 of FlhA is critical for PMF-driven protein export and that most of substitutions are tolerated by the presence of FlhB, FliH and FliI. We also show an interaction between FlhA and FliR.

## Results

### Alanine mutagenesis of conserved charged residues of FlhATM

**Figure 2:**
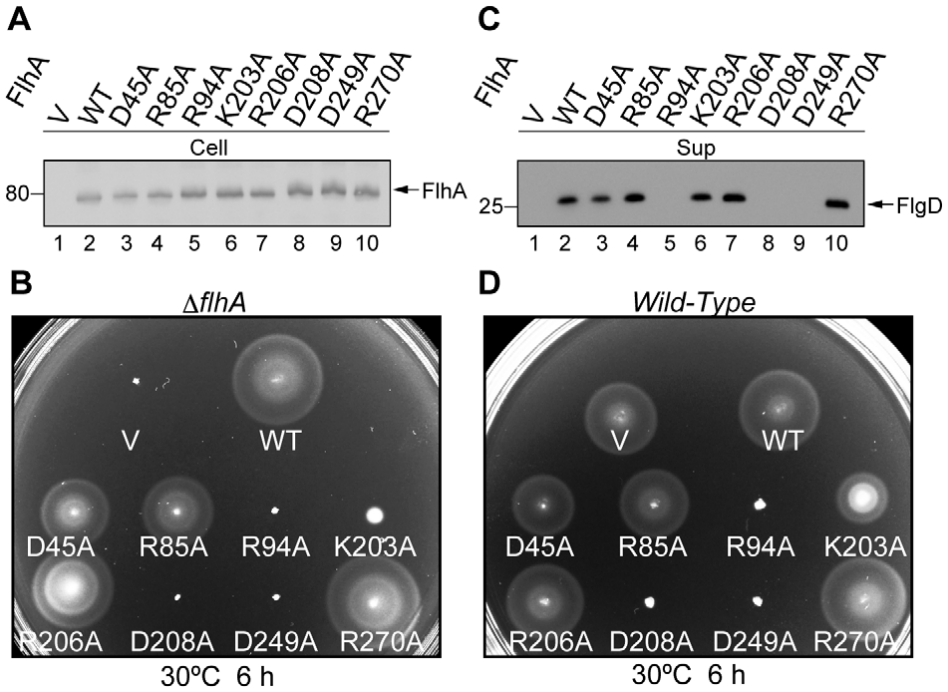
Effect of alanine substitutions in FlhATM on motility and flagellar protein export. (A) Expression levels of alanine-substituted variants of FlhA in cells. Immunoblotting, using polyclonal anti-FlhAC antibody, of whole cells protein (Cell) prepared from a flhA null strain, NH0001 (ΔflhA), transformed with pUC19-based plasmids encoding various alanine-substituted forms of FlhA. V, pUC19; WT, wild-type FlhA; D45A, FlhA(D45A); R85A, FlhA(R85A); R94A, FlhA(R94A); K203A, FlhA(K203A); R206A, FlhA(R206A); D208A, FlhA(D208A); D249A, FlhA(D249A); R270A, FlhA(R270A). (B) Motility of the flhA null mutant transformed with the above plasmids in soft agar. The plate was incubated at 30°C for 6 hours. (C) Secretion of FlgD. Immunoblotting, using polyclonal anti-FlgD antibody, of culture supernatants (Sup) prepared from the above strains. (D) Dominant negative effect on motility of the wild-type strain SJW1103 in soft agar. The plate was incubated at 30°C for 6 hours.

To test the hypothesis that highly conserved charged residues of FlhATM is involved in the energy transduction mechanism, we identified eight highly conserved charged residues, Asp-45, Arg-85, Arg-94, Lys-203, Arg-206, Asp-208, Asp249 and Arg-270, in putative juxta- and trans-membrane helices of FlhA by multiple sequence alignment (Figures 1 and S2) and then replaced each with alanine. Immunoblotting with polyclonal anti-FlhAC antibody detected all the point mutant variants at the wild-type level (Figure 2A), indicating that these substitutions do not affect protein stability.

We next analyzed the motility of the flhA null mutant expressing each of the FlhA point mutant variants in soft agar plates (Figure 2B). FlhA(R206A) and FlhA(R270A) fully complemented, FlhA(D45A) and FlhA(R85A) restored motility to a significant degree, and FlhA(K203A) to some degree. However, mutants with alanine substitution for Arg-94, Asp-208 and Asp-249 did not complement at all. To test whether their poor motility is due to their reduced export activity, we prepared the culture supernatants from the overnight culture of these flhA point mutants and analyzed the secretion levels of the hook-capping protein FlgD by immunoblotting with polyclonal anti-FlgD antibody (Figure 2C). FlgD was detected at the wild-type level in the culture supernatants from the D45A, R85A, K203A, R206A, and R270A mutants but not from the R94A, D208A, and D249A mutants. FlhA(R94A), FlhA(D208A) and FlhA(D249A) also inhibited motility when expressed in the wild-type strain (Figure 2D), indicating that these mutant proteins exert a negative dominance. This suggests that they can be incorporated into the export apparatus. Therefore, we conclude that Arg-94, Asp-208, and Asp-249 are critical for protein export.

### Importance of the charge of critical residues

**Figure 3:**
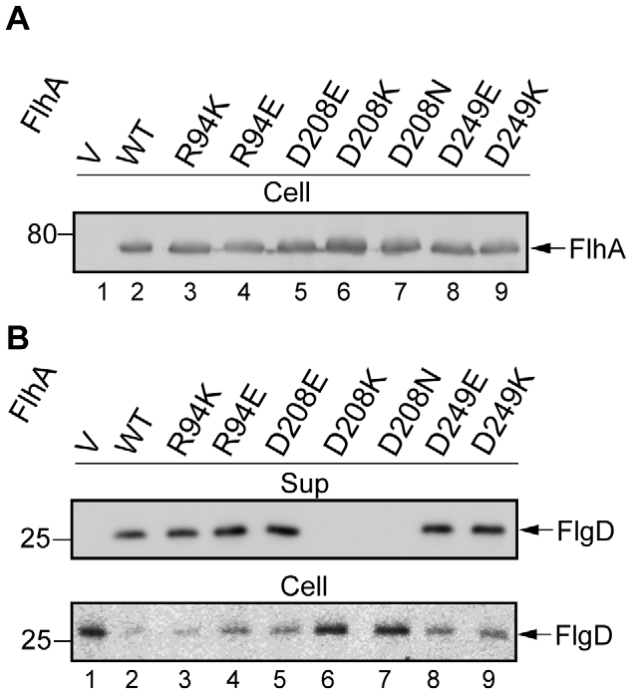
Absolute requirement of a negatively charged residue at position 208 of FlhA. (A) Expression levels of the mutant FlhA proteins. Immunoblotting, with polyclonal anti-FlhAC antibody, of a flhA null strain transformed with pUC19-based plasmids encoding various forms of FlhA. V, pUC19; WT, wild-type FlhA; R94K, FlhA(R94K); R94E, FlhA(R94E); D208E, FlhA(D208E); D208K, FlhA(D208K); D208N, FlhA(D208N); D249E, FlhA(D249E); D249K, FlhA(D249K). (B) Secretion of FlgD. Immunoblotting, using polyclonal anti-FlgD antibody of whole cell (Cell) and culture supernatant (Sup) fractions prepared from the above strains.

To probe the role of Arg-94, Asp-208 and Asp-249 in protein export, we mutated these three residues to the following two types: the same charge with a different length of side chain (Arg-to-Lys and Asp-to-Glu); and the oppositely charged residue (Arg-to-Asp and Asp-to-Lys). Immunoblotting with the polyclonal anti-FlhAC antibody revealed that all the mutant variants were as stable as the wild-type (Figure 3A). At positions 94 and 249, neither types of mutations affected the secretion level of FlgD (Figure 3B, lanes 3, 4, 8 and 9), indicating that these residues maintain the function of FlhA regardless of the charge type. While the D208E replacement still permitted the export of FlgD at the wild-type level (Figure 3B, lane 5) the D208K and D208N mutations totally diminished the export (lanes 6 and 7). In agreement with these results, the D208K and D208N mutants as well as the flhA null mutant harboring the vector control accumulated much higher amounts of FlgD in the cytoplasm than the wild-type while the others did so more or less at the wild-type levels. FlhA(D208K) and FlhA(D208N) also exerted a dominant negative effect on wild-type motility (data not shown), indicating that they retain the ability to be incorporated into the export apparatus. These results suggest that a negatively charged residue at position 208 of FlhA is essential for the export function.

### Protein secretion rate of slow motile mutants

**Figure 4:**
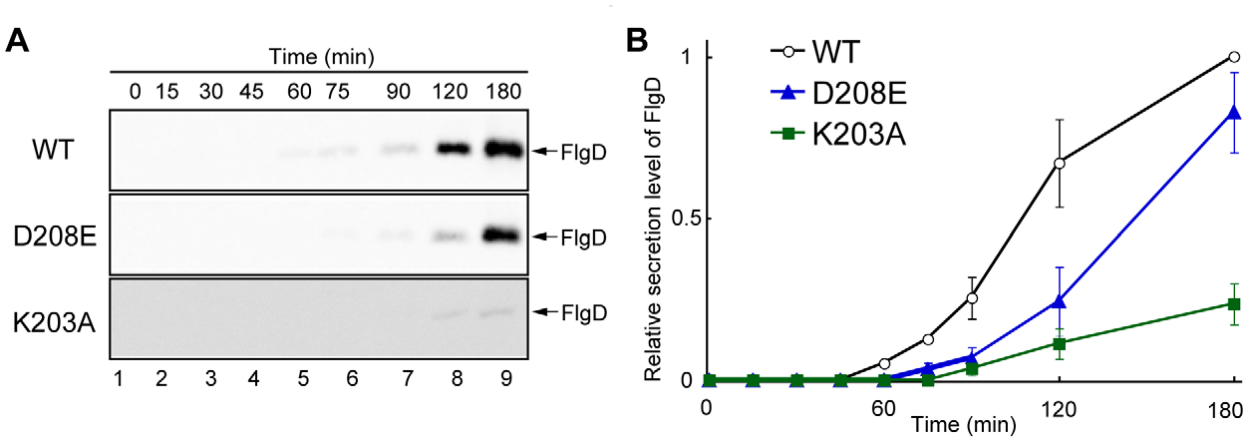
FlgD secretion rate of slow motile flhA mutants. (A) Immunoblotting, using polyclonal anti-FlgD antibody, of culture supernatants prepared from NH0005 (ΔflhA PflhDC::T-POP(DEL-25) transformed with pMMHA004 (wild-type FlhA, WT), pNH001(D208E) (FlhA(D208E), D208E) or pNH001(K203A) (FlhA(K203A), K203A). After adding tetracycline, culture supernatants were collected at 0, 15, 30, 45, 60, 75, 90, 120 and 180 min and analyzed with polyclonal anti-FlgD antibody. (B) Relative secretion levels of FlgD, normalized for the level of FlgD in wild-type cells at 180 min. Shown are the mean and standard deviation of three independent experiments.

We found that the motility of the flhA(D208E) (data not shown) and flhA(K203A) (Figure 2B) mutants was worse than that of wild-type cells in soft agar plates whereas the levels of FlgD secretion by these mutants were at the wild-type level when grown overnight in LB (Figure 3B, lane 5, and Figure 2C, lane 6, respectively). These results raise the possibility that these mutants are slow secretors. Secretion rate measurement of the flagellar proteins requires the external onset control of flagellar gene expression. To do this, we inserted a Tn10d (T-POP) transposon upstream of the flagellar master flhDC operon, which is required for the expression of the entire flagellar regulon. As flhDC is transcribed from a tetracycline-inducible promoter PtetA only in the presence of tetracycline, flagellar gene expression can be externally controlled [34]. A flhA null mutant containing T-POP was transformed with pUC19-based plasmids encoding wild-type FlhA or each of the FlhA mutants. The transformants were grown at 30°C in LB until OD600 reached ca. 0.5∼0.6. After induction with tetracycline, the culture supernatants were collected at regular time intervals and analyzed by immunoblotting with polyclonal anti-FlgD antibody (Figure 4).

For wild-type FlhA, FlgD was detected at 60 min after induction and the amount of FlgD gradually increased as the incubation was continued. The D208E mutation caused a 15 min delay in the detection of FlgD. In agreement with this, when comparing the length of flagellar filaments labeled with a fluorescent dye, the filaments of these mutants were significantly shorter than those of the wild-type at 120 minutes after tetracycline induction (data not shown). When the incubation was further continued for 12 hours, the filaments grew nearly to the wild-type level. These results suggest that the D208E mutation reduces the rate of protein export. For the flhA(K203A) mutant, FlgD was not detected until 120 min after induction, indicating that this is a much slower secretor than the flhA(D208E) mutant.

### Effect of flhA mutations on flagellar protein export in the ΔfliH-fliI double null mutant background

**Figure 5:**
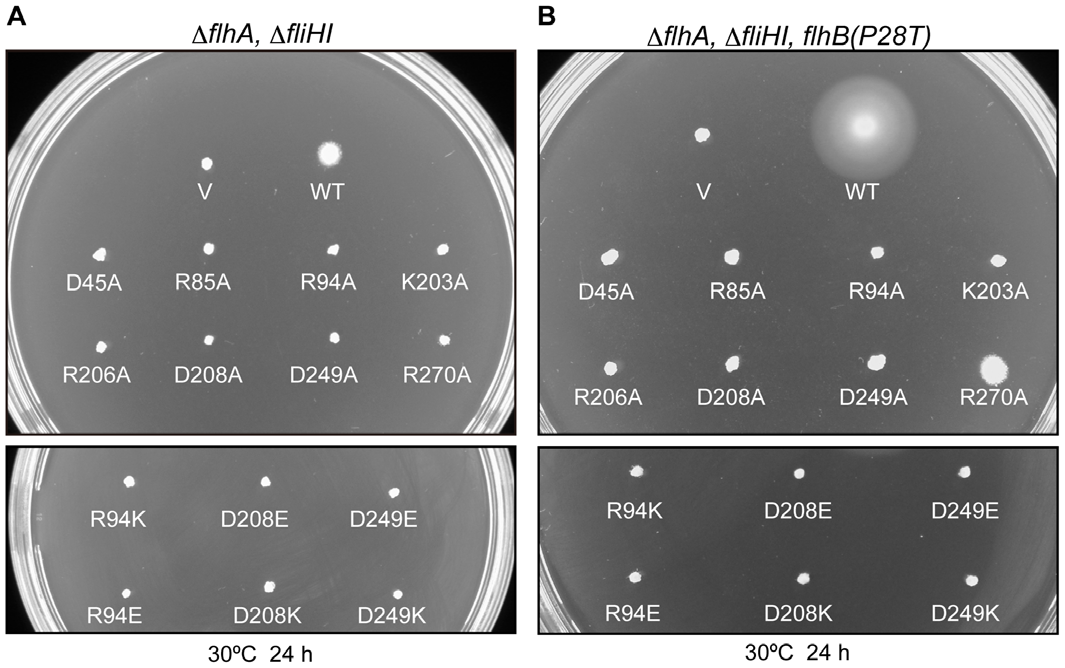
Effect of a ΔfliH-fliI mutation and its bypass mutation FlhB(P28T) on FlhA mutants. (A) Motility of the NH0003 (ΔfliH-fliI ΔflhA) and (B) the NH0004 [ΔfliH-fliI flhB(P28T) ΔflhA] mutant strains transformed with pUC19-based plasmids encoding various forms of FlhA in soft agar. V, pUC19; WT, wild-type FlhA; D45A, FlhA(D45A); R85A, FlhA(R85A); R94A, FlhA(R94A); K203A, FlhA(K203A); R206A, FlhA(R206A); D208A, FlhA(D208A); D249A, FlhA(D249A); R270A, FlhA(R270A); R94K, FlhA(R94K); R94E, FlhA(R94E); D208E, FlhA(D208E); D208K, FlhA(D208K); D208N, FlhA(D208N); D249E, FlhA(D249E); D249K, FlhA(D249K).

It has been previously reported that a ΔfliH-fliI double null mutant is weakly motile [22]. We tested if the flhA mutations that did not affect protein export are also tolerated in the absence of FliH and FliI (Figure 5). The weakly motile phenotype of the ΔfliH-fliI mutant was totally abolished by the flhA mutations (Figure 5A), indicating that these mutant FlhA proteins have a more synergistic phenotype when combined with the flH-fliI double null mutation.

**Figure 6:**
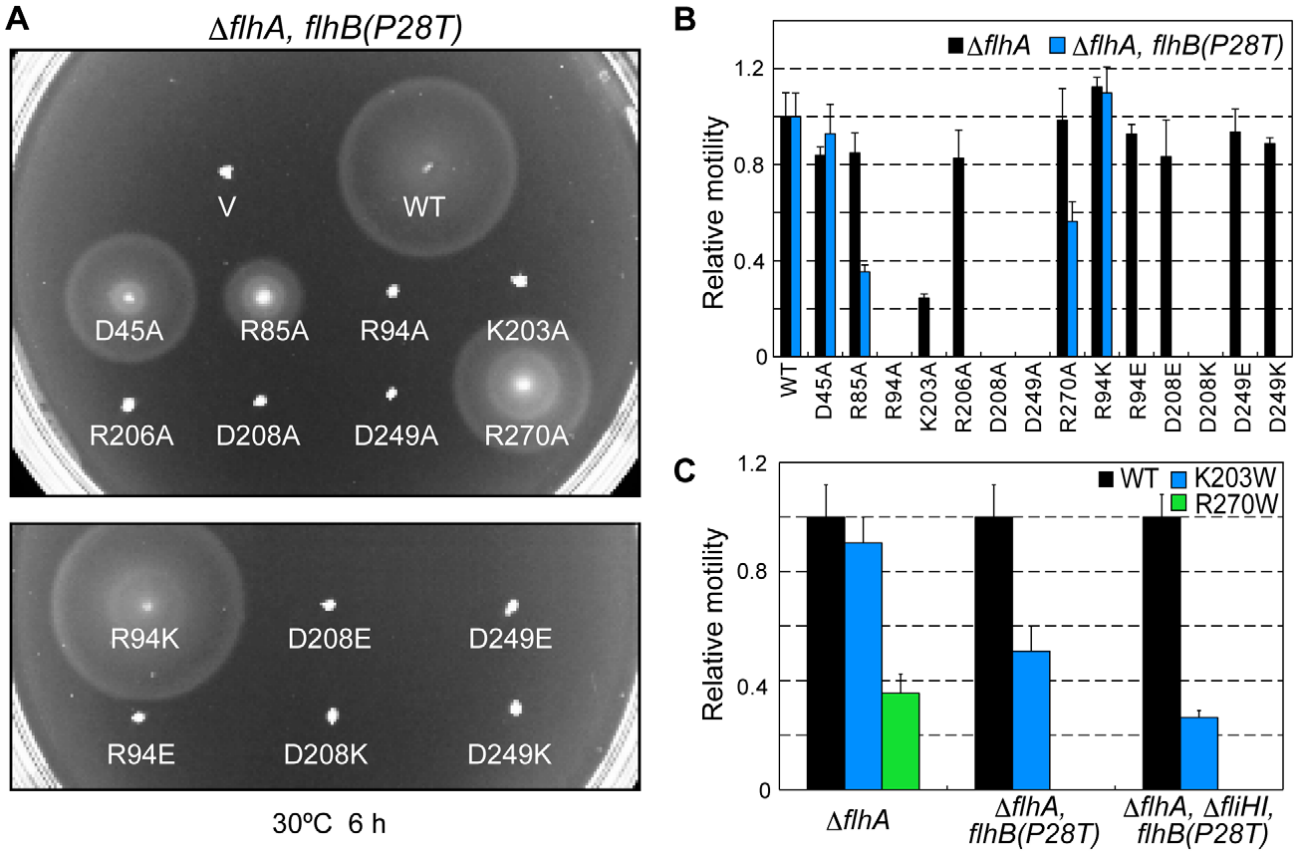
Effect of the FlhB(P28T) mutation alone on FlhA mutants. (A) Motility of the NH0002 [ΔflhA flhB(P28T)] mutant strain transformed with pUC19-based plasmids encoding various forms of FlhA in soft agar. V, pUC19; WT, wild-type FlhA; D45A, FlhA(D45A); R85A, FlhA(R85A); R94A, FlhA(R94A); K203A, FlhA(K203A); R206A, FlhA(R206A); D208A, FlhA(D208A); D249A, FlhA(D249A); R270A, FlhA(R270A); R94K, FlhA(R94K); R94E, FlhA(R94E); D208E, FlhA(D208E); D208K, FlhA(D208K); D208N, FlhA(D208N); D249E, FlhA(D249E); D249K, FlhA(D249K). (B) Relative motility of those shown in (A). The mean and standard deviation of three independent measurements of the diameter of each motility ring relative to that formed by the wild-type control (strain NH0001 or NH0002 harboring pMMHA004). (C) Relative motility of tryptophan-substituted FlhA mutants in soft agar. The mean and standard deviation are calculated from three independent measurements of motility ring size of NH0001, NH0002 and NH0004 containing FlhA mutants relative to each strains harboring pMMHA004.

Minamino & Namba [22] isolated a ΔfliH-fliI bypass mutant whose second-site P28T mutation in FlhB significantly increases the probability of substrate entry into the gate in the absence of FliH and FliI. Therefore, we tested if the mutant FlhA proteins can work in this ΔfliH-fliI bypass mutant (Figure 5B). Wild-type FlhA restored the motility of the ΔfliH-fliI flhB(P28T) ΔflhA mutant, in agreement with a previous report [22]. However, only FlhA(R270A) complemented this mutant to some degree although markedly less than the wild-type level, and the others did not show any complementation at all. We next investigated the effect of the flhB(P28T) mutation by itself on the function of these mutant FlhA proteins. In agreement with a previous report [22], wild-type FlhA fully restored the motility of a ΔflhA flhB(P28T) mutant (Figure 6A). The presence of FliH and FliI allowed FlhA(D45A), FlhA(R85A), FlhA(R270A) and FlhA(R94K) to exert their function considerably even in the presence of the flhB(P28T) mutation but none of the remaining mutants (Figure 6A and B). These results indicate a degree of defectiveness for the individual flhA alleles.

Tryptophan mutation can probe the structure and environment of transmembrane domains of membrane proteins [35]–[37]. We performed tryptophan mutagenesis to test if the ΔfliH-fliI bypass flhB(P28T) mutation and the FliH-FliI complex affect the structural architecture of FlhATM. The flhA(K203W) and flhA(R270W) mutants in the otherwise wild-type background remained functional mostly and to some degree, respectively, while the others were nonfunctional (Figure S3). Since tryptophan substitution for residues facing the lipid preserves the function but is likely to disrupt the structure and function for residues within or adjacent to transmembrane segments, the results indicate that Lys-203 and Arg-270 of FlhA are likely to face the lipid or water-exposed channel of the export gate complex. We then analyzed the effect of the K203W and R270W mutations on motility of the ΔfliH-fliI flhB(P28T) mutant and the flhB(P28T) mutant. The motility of these mutants was significantly reduced compared with the wild-type (Figure 6C). Since Lys-203 and Arg-270 are predicted to be located in the conserved cytoplasmic loop between TM-4 and TM-5 and within TM-6, respectively (Figure 1), these residues may face the interior of the gate complex in the presence of the flhB(P28T) mutation.

### Isolation of pseudorevertants from the ΔfliH-fliI flhB(P28T) flhA(K203W) mutant

**Figure 7:**
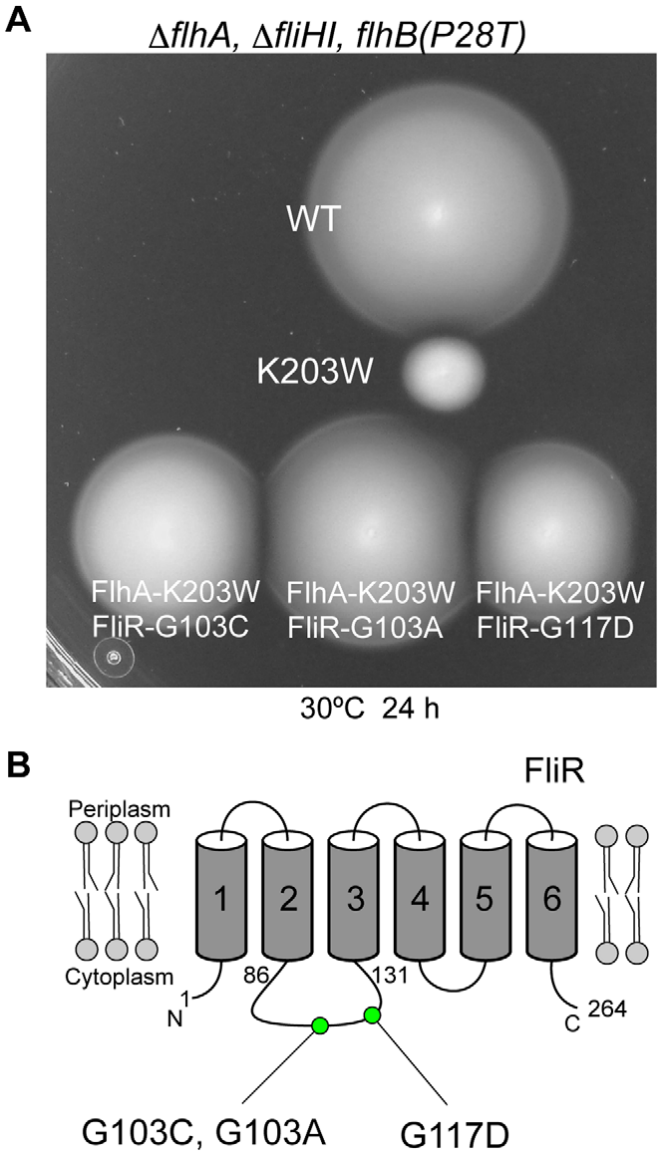
Isolation of pseudorevertants from the flhA(K203W) mutant. (A) Motility of NH0004 expressing wild-type FlhA or FlhA(K203W), and NH0007 [ΔfliH-fliI ΔflhA flhB(P28T) fliR(G103C)], NH0008 [ΔfliH-fliI ΔflhA flhB(P28T) fliR(G103A)] and NH0009 [ΔfliH-fliI ΔflhA flhB(P28T) fliR(G117D)] harboring the K203W mutant in soft agar plate. (B) Positions of flhA(K203W) suppressor mutations in FliR.

To investigate how the K203W mutation significantly reduces the export activity in the ΔfliH-fliI bypass mutant, gain-of-function mutants were isolated from the ΔfliH-fliI flhB(P28T) flhA(K203W) mutant. In total three pseudorevertants were obtained with significantly improved motility although not as good as that of the wild-type (Figure 7A). DNA sequencing revealed that the second-site mutations are located in FliR, and they were G103A, G103C and G117D (Figure 7B). These mutated residues are not conserved among FliR homologs (Figure S4). They were all located in a cytoplasmic loop between TM-2 and TM-3, suggesting that the conserved loop between TM-4 and TM-5 in FlhA may interact with the cytoplasmic loop of FliR.

To test whether these extragenic fliR suppressors are allele specific for the parental flhA(K203W) mutation, we carried out P22-mediated transductional crosses. The ΔfliH-fliI ΔflhA flhB(P28T) fliR::Tn10 strain harboring the pUC19-based plasmid encoding each mutant FlhA protein was used as a recipient and the three pseudorevertants isolated were used as donors. A 50 µl solution of the overnight culture of the recipient and a 5 µl aliquot of phage lysates prepared from the donors were mixed together and then streaked on soft agar plates. After 40 hours incubation at 30°C, we looked for motility halos resulting from homologus recombination between the fliR::Tn10 and the suppressor fliR alleles. Motility halos appeared from the recipient expressing FlhA(K203W) but not from those expressing FlhA(K203A) or FlhA(R270W) as shown by the data of the fliR(G103A) allele (Figure S5A). These results indicate that these fliR suppressors display allele specificity. This suggests an interaction between the conserved cytoplasmic loop of FlhA and the cytoplasmic loop of FliR

To test the effect of each second-site fliR mutation by itself on motility, we isolated the second-site fliR mutants and analyzed their motility on soft agar plates. In all cases motility was nearly normal, just slightly less than the wild-type (Figure S5B), indicating that they do not affect the function of FliR significantly.

## Discussion

FlhA_C_ not only acts as part of the docking platform of the export gate complex for the soluble export components, FliH, FliI, and FliJ, but also plays a critical role in PMF-driven flagellar protein export along with FliH, FliI, FliJ and FlhB_C_ [23]. However, little is known about the role of FlhA_TM_ in PMF-driven flagellar protein export. Here, we carried out genetic analysis of eight highly conserved charged residues of FlhA_TM_ and provide evidence suggesting that Asp-208 is critical for the translocation of export substrate and that the cooperative interactions of FlhA with FlhB, FliH, FliI, and FliR facilitates PMF-driven flagellar protein export.

### Asp-208 of FlhA is essential for flagellar protein export

Two conserved charged residues of *E. coli* F_O_F_1_-ATP synthase, Arg-210 of the a subunit and Asp-61 of the c subunit, are critical for proton flow though F_O_, which is coupled with ATP synthesis/hydrolysis by F_1_ [38]–[40]. Asp-61 is directly involved in proton translocation [39]. Arg-210 is essential for preventing the short-circuiting of proton flow without c-ring rotation in the F_O_ proton channel [40]. Since there are many similarities between the flagellar type III protein export apparatus and F_O_F_1_-ATP synthase [3], the highly conserved acidic and basic residues of the transmembrane components of the flagellar export apparatus may be directly involved in the inwardly-directed proton flow. If so, the function of putative proton-binding sites in the export gate is presumably critical. In this study, we probed the function of highly conserved charged residues in trans- or juxta-transmembrane helices of FlhA by mutating these charged residues and found that only Asp-208 of FlhA is critical for the functioning of the export apparatus (Figure 2 and 3). The D208A, D208W, D208K, and D208N mutant alleles were dominant to the wild-type *flhA* gene and hence totally inhibited motility of wild-type cells. This suggests that these mutants retain the ability to be incorporated into the export apparatus. Because only the conservative D208E replacement permitted any function, the important feature of this residue appears to be either a negative side-chain charge or the ability to bind a proton. Asp-208 of FlhA is predicted to be located in the cytoplasmic juxtamembrane region (Figure 1), whereas both Arg-210 of a subunit and Asp-61 of c subunit are located at the center of a transmembrane helix. This suggests that Asp-208 in FlhA may not directly be involved in the proton translocation mechanism. But since Asp-208 is involved in PMF-driven flagellar protein export, we assume that a negative side-chain charge of Asp-208 may be required for an electrostatic interaction with other export component(s) and/or export substrates.

### A degree of defectiveness for the individual flhA alleles is dependent on the presence of FlhB, FliH and FliI

In this study, we found that most of substitutions of evolutionally conserved charged residues except for Asp-208 were tolerated when FliH and FliI were present. In contrast, these substitutions totally abolished a weakly motile phenotype of a ΔfliH-fliI double null mutant. The P28T mutation in FlhB increases the probability of export substrate entry into the narrow pore of the export gate in the absence of FliH and FliI [22]. Here, we also found that those FlhA mutants did not function in the ΔfliH-fliI flhB(P28T) bypass mutant (Figure 5B). FliH and FliI overcame the D45A, R85A, R94K and R270A defects even in the presence of the *flhB*(P28T) allele (Figure 6A), indicating that the *flhB*(P28T) significantly affects the function of these mutant FlhA proteins and that FliH and FliI restore their function. Taken all together we suggest that these mutant FlhA proteins require the FliH-FliI complex and FlhB to exert their export function.

Tryptophan substitution at positions 203 and 270 in FlhATM significantly reduced the export activity when the FlhB(P28T) mutation was present (Figure 6C). Tryptophan substitution is assumed to preserve the protein function when the mutated residues face lipid or water but to disrupt structure and function when the residues face adjacent protein segments [35]–[37]. Mutant FlhA(K203W) and FlhA(R270W) proteins retained the FlhA function when FlhB, FliH, and FliI were present (Figure S3), indicating that the orientation of the side-chain of these tryptophan residues is significantly affected by FlhB, FliH and FliI. Therefore, we propose that cooperative interactions among FlhA, FlhB, FliH, FliI and export substrate may induce conformational changes in FlhATM to facilitate PMF-driven protein export.

### Interaction of the well-conserved cytoplasmic loop between TM-4 and TM-5 of FlhA with a cytoplasmic loop of FliR

A highly conserved hydrophilic cytoplasmic loop is located between TM-4 and TM-5 of FlhA, but little is known about its role in flagellar protein export [27]. In this study, we found that the reduced motility of the ΔfliH-fliI flhB(P28T) flhA(K203W) mutant was significantly improved by extragenic mutations in the cytoplasmic loop between TM-2 and TM-3 of FliR (Figure 7). Interestingly, these suppressors were allele specific for the flhA(K203W) mutation, suggesting an interaction between the conserved cytoplasmic loop of FlhA and the cytoplasmic loop of FliR. The FliR-FlhB fusion protein is partially functional, suggesting that FliR interacts with FlhB [13]. Therefore, it is likely that FlhA, FlhB and FliR are very close to each other in the export gate complex. This is supported by our finding that the P28T bypass mutation, which is close to the interface of TM-1 of FlhB, significantly affects the function of mutant FlhA.

The K203A mutation considerably reduced the secretion rate of FlgD in a way similar to the D208E mutation (Figure 4). Since Lys-203 is likely to be close to Asp-208, we propose that the precise conformation of a negatively charged residue at position 208 of FlhA may be important for protein export and that the interaction of the conserved cytoplasmic loop of FlhA with the cytoplasmic loop of FliR is required for maintaining this conformation.

## Materials and Methods

### Bacterial strains, plasmids and media

Bacterial strains and plasmids used in this study are listed in Table 1. L-broth and soft tryptone agar plates were used as described previously [9], [10]. Tetracycline-sensitive (Tc^S^) plates, in which the tetracycline-resistant cells cannot grow, were prepared as described by Maloy and Nunn [41]. Ampicillin and tetracycline were added to LB at a final concentration of 100 µg/ml and 15 µg/ml, respectively.

**Table 1:** Strains and Plasmids used in this study.

Strains and Plasmids	Relevant characteristics	Source or reference
*Salmonella*		
SJW1103	Wild type for motility and chemotaxis	[42]
NH0001	Δ*flhA*	This study
NH0002	Δ*flhA flhB*(P28T)	This study
NH0003	Δ*fliH-fliI* Δ*flhA*	This study
NH0004	Δ*fliH-fliI* Δ*flhA flhB*(P28T)	This study
NH0005	Δ*flhA* P*_flhDC_*::T-POP(DEL-25)	This study
NH0006	Δ*fliH-fliI* Δ*flhA flhB*(P28T) *fliR*::Tn*10*	This study
NH0007	Δ*fliH-fliI* Δ*flhA flhB*(P28T) *fliR*(G103C)/pNH001(K203W)	This study
NH0008	Δ*fliH-fliI* Δ*flhA flhB*(P28T) *fliR*(G103D)/pNH001(K203W)	This study
NH0009	Δ*fliH-fliI* Δ*flhA flhB*(P28T) *fliR*(G117D)/pNH001(K203W)	This study
NH0010	*fliR*(G103C)	This study
NH0011	*fliR*(G103D)	This study
NH0012	*fliR*(G117D)	This study
Plasmids		
pUC19	Cloning vector	Invitrogen
pMMHA004	pUC19/His-FlhA	[45]
pNH001(D45A)	pUC19/His-FlhA(D45A)	This study
pNH001(R85A)	pUC19/His-FlhA(R85A)	This study
pNH001(R94A)	pUC19/His-FlhA(R94A)	This study
pNH001(K203A)	pUC19/His-FlhA(K203A)	This study
pNH001(R206A)	pUC19/His-FlhA(R206A)	This study
pNH001(D208A)	pUC19/His-FlhA(D208A)	This study
pNH001(D249A)	pUC19/His-FlhA(D249A)	This study
pNH001(R270A)	pUC19/His-FlhA(R270A)	This study
pNH001(D45W)	pUC19/His-FlhA(D45W)	This study
pNH001(R85W)	pUC19/His-FlhA(R85W)	This study
pNH001(R94W)	pUC19/His-FlhA(R94W)	This study
pNH001(K203W)	pUC19/His-FlhA(K203W)	This study
pNH001(R206W)	pUC19/His-FlhA(R206W)	This study
pNH001(D208W)	pUC19/His-FlhA(D208W)	This study
pNH001(D249W)	pUC19/His-FlhA(D249W)	This study
pNH001(R270W)	pUC19/His-FlhA(R270W)	This study
pNH001(R94K)	pUC19/His-FlhA(R94K)	This study
pNH001(R94E)	pUC19/His-FlhA(R94E)	This study
pNH001(D208E)	pUC19/His-FlhA(D208E)	This study
pNH001(D208K)	pUC19/His-FlhA(D208K)	This study
pNH001(D208N)	pUC19/His-FlhA(D208N)	This study
pNH001(D249E)	pUC19/His-FlhA(D249E)	This study
pNH001(D249K)	pUC19/His-FlhA(D249K)	This study

### Transductional crosses and DNA manipulations

P22-mediated transductional crosses were carried out using p22*HTint* as described previously [42]. DNA manipulations were carried out as described before [10]. Site-directed mutagenesis was carried out using QuickChange site-directed mutagenesis method as described in the manufacturer's instructions (Stratagene) with a plasmid, pMMHA004 (pUC19/His-FlhA), as a template DNA. The pairs of oligonucleotides shown in Table 2 were used to construct each FlhA mutant. All of the *flhA* mutations were confirmed by DNA sequencing. DNA sequencing reactions were carried out using BigDye v3.1 as described in the manufacturer's instructions (Applied Biosystems), and then the reaction mixtures were analyzed by a 3130 Genetic Analyzer (Applied Biosystems).

**Table 2:** Primers used in this study.

Mutated Residue	Primer	Sequence (5′-3′)
D45A	F	CCGCTACCTGCTTTTATCCTCGCCTTATTGTTTACCTTTAATATT
	R	AATATTAAAGGTAAACAATAAGGCGAGGATAAAAGCAGGTAGCGG
R85A	F	GTTTACCACGCTACTGGCTCTGGCGCTTAACGTTG
	R	CAACGTTAAGCGCCAGAGCCAGTAGCGTGGTAAAC
R94A	F	CTTAACGTTGCCTCAACGGCCATTATTTTGATGGAAGGG
	R	CCCTTCCATCAAAATAATGGCCGTTGAGGCAACGTTAAG
K203A	F	GGACGGGGCAAGTGCATTTGTACGCGGCG
	R	CGCCGCGTACAAATGCACTTGCCCCGTCC
R206A	F	GCAAGTAAATTTGTAGCCGGCGACGCCATCGCC
	R	GGCGATGGCGTCGCCGGCTACAAATTTACTTGC
D208A	F	AAATTTGTACGCGGCGCCGCCATCGCCGGTATTC
	R	GAATACCGGCGATGGCGGCGCCGCGTACAAATTT
D249A	F	CTGACCATTGGCGCCGGCCTGGTCGC
	R	GCGACCAGGCCGGCGCCAATGGTCAG
R270A	F	GGCGTCATTGTGACCGCCGTTAGTACCGATCAG
	R	CTGATCGGTACTAACGGCGGTCACAATGACGCC
D45W	F	CCGCTACCTGCTTTTATCCTCTGGTTATTGTTTACCTTTAATATT
	R	AATATTAAAGGTAAACAATAACCAGAGGATAAAAGCAGGTAGCGG
R85W	F	CTGTTTACCACGCTACTGTGGCTGGCGCTTAACGTTGCC
	R	GGCAACGTTAAGCGCCAGCCACAGTAGCGTGGTAAACAG
R94W	F	GCGCTTAACGTTGCCTCAACGTGGATTATTTTGATGGAAGGGCAT
	R	ATGCCCTTCCATCAAAATAATCCACGTTGAGGCAACGTTAAGCGC
K203W	F	ATGGACGGGGCAAGTTGGTTTGTACGCGGCGAC
	R	GTCGCCGCGTACAAACCAACTTGCCCCGTCCAT
R206W	F	GCAAGTAAATTTGTATGGGGCGACGCCATCGCC
	R	GGCGATGGCGTCGCCCCATACAAATTTACTTGC
D208W	F	AGTAAATTTGTACGCGGCTGGGCCATCGCCGGTATTCTC
	R	GAGAATACCGGCGATGGCCCAGCCGCGTACAAATTTACT
D249W	F	CTGCTGACCATTGGCTGGGGCCTGGTCGCCCAG
	R	CTGGGCGACCAGGCCCCAGCCAATGGTCAGCAG
R270W	F	GCGGGCGTCATTGTGACCTGGGTTAGTACCGATCAGGAT
	R	ATCCTGATCGGTACTAACCCAGGTCACAATGACGCCCGC
R94K	F	GGCGCTTAACGTTGCCTCAACGAAGATTATTTTGATGGAAGGGC
	R	GCCCTTCCATCAAAATAATCTTCGTTGAGGCAACGTTAAGCGCC
R94E	F	CGCTTAACGTTGCCTCAACGGAGATTATTTTGATGGAAGGGC
	R	GCCCTTCCATCAAAATAATCTCCGTTGAGGCAACGTTAAGCG
D208E	F	AAATTTGTACGCGGCGAGGCCATCGCCGGTATTC
	R	GAATACCGGCGATGGCCTCGCCGCGTACAAATTT
D208K	F	AAATTTGTACGCGGCAAGGCCATCGCCGGTATTC
	R	GAATACCGGCGATGGCCTTGCCGCGTACAAATTT
D208N	F	AAATTTGTACGCGGCAACGCCATCGCCGGTATTC
	R	GAATACCGGCGATGGCGTTGCCGCGTACAAATTT
D249E	F	CTGACCATTGGCGAGGGCCTGGTCGC
	R	GCGACCAGGCCCTCGCCAATGGTCAG
D249K	F	GCTGACCATTGGCAAGGGCCTGGTCGCC
	R	GGCGACCAGGCCCTTGCCAATGGTCAGC

For site-directed mutagenesis of FlhA.

F, forward; R, reverse.

### Construction of the *flhA* null strains

To delete the *flhA* gene form the chromosome, we used the λ Red homologous recombination system developed by Datsenko and Wanner [43]. First, the entire *flhA* gene was replaced with the *tetRA* genes to construct a Δ*flhA*::*tetRA* strain as described [44]. Then, to remove the *tetRA* genes from the chromosome, the 112-bp DNA was first synthesized and then was purified using a QIAquick PCR purification kit (QIAGEN). The Δ*flhA*::*tetRA* strain carrying pKD46, which has a temperature sensitive replicon [43], was grown in 5-ml L-broth containing ampicillin and 0.2% L-arabinose at 30°C until OD_600_ had reached 0.6. The cells were washed three times with ice-cold H_2_O and suspended in 50 µl of ice-cold H_2_O. 50 µl of cells were electroporated with 100 to 200 ng of purified 112-bp DNA using 0.1-cm cuvettes at 1.8 kV. Shocked cells were added to 1 ml SOC, and incubated for 1 h at 37°C. Then, one-half were spread onto Tc^S^ plates and incubated at 42°C overnight. The constructs were conformed by DNA sequencing.

### Motility assay

Fresh transformants were inoculated onto soft tryptone agar plates containing ampicillin and incubated at 30°C.

### Preparation of whole cell and culture supernatant fractions and immunoblotting

*Salmonella* cells were grown at 30°C with shaking until the cell density had reached an OD_600_ of ca. 1.4–1.6. Aliquots of culture proteins containing a constant number of cells were clarified by centrifugation. Cell pellets were resuspended in SDS-loading buffer normalized by cell density to give a constant amount of cells. The proteins in the culture supernatants were precipitated by 10% trichloroacetic acid (TCA), suspended in a Tris-SDS loading buffer and heated at 95°C for 5 min. After sodium dodecyl sulfate-polyacrylamide gel electrophoresis (SDS-PAGE), immunoblotting with polyclonal anti-FlgD or anti-FlhA_C_ antibody was carried out as described previously [9]. Detection was done with an ECL immunoblotting detection kit (GE Healthcare).

### Tetracycline-induced protein export rate assay

*Salmonella* cells with a T-POP insertion between the *flhDC* promoter and the transcription start site [34], were grown overnight in LB culture at 30°C and diluted 50-fold into 30 ml of fresh LB. When the cell density reached an OD_600_ of 0.5, tetracycline was added to a final concentration of 15 µg/ml. Samples were taken at 0, 15, 30, 45, 60, 75, 90, 120 and 180 min after tetracycline addition. Proteins in the culture supernatant fraction were prepared by TCA precipitation as described above.

### Multiple sequence alignment

PSI-BLAST searches were initiated on the NCBI's BLAST server (http://www.ncbi.nlm.nih.gov/BLAST/) under default conditions to obtain FlhA homologs. Multiple sequence alignment was done by CLUSTAL-W (http://clustalw.ddbj.nig.ac.jp/top-j.html).

## Supporting Information

Figure S1 **Schematic diagram of the bacterial flagellar type III protein export apparatus.** The flagellar type III protein export apparatus consists of three soluble proteins, FliH, FliI, and FliJ and six integral membrane proteins, FlhA, FlhB, FliO, FliP, FliQ, and FliR. The integral membrane proteins are postulated to be located within the central pore of the MS ring and form the PMF-driven export gate complex. FlhA and FlhB have large cytoplasmic domains which project into the cavity within the C ring and form the docking platform for FliH, FliI, and FliJ as well as the substrates. FliI forms a heterotrimer with the FliH dimer in the cytoplasm. The FliH2FliI complex, along with FliJ and export substrates, is localized to the basal body C-ring through a specific interaction between FliH and FliN. FliI hexamerizes upon docking of the FliH-FliI-FliJ-substrate complex to the FlhA-FlhB platform and facilitates the entry of the N-terminal segment of a substrate into the gate. ATP hydrolysis by the FliI hexamer induces the dissociation of the FliHX-FliI6-FliJ complex from the gate. The export gate utilizes PMF across the cytoplasmic membrane as the energy source for the translocation of the export substrates into the central channel of the growing flagellar structure.
(TIF)

Figure S2 **Multiple sequence alignment of FlhA homologs.** Multiple sequence alignment was carried out by CLUSTAL-W (http://clustalw.ddbj.nig.ac.jp/top-j.html). Green boxes encircle putative transmembrane domains. UniProt Accession numbers: Salmonella (P40729); Escherichia (P76298); Yersinia (O56887); Pseudomonas (Q4KG43); Aquifex (O67265); Caulbacter (Q03845); Vibrio (Q9Z6F4); Bacillus (Q03845); Helicobacter (O06758); InvA_Salmonella (P0A1I3); LcrD_Yersinia (P66655); SsaV_Salmonella (P74856). Red and blue shades stars indicate conserved acidic and basic residues, respectively, which are selected for site-directed mutagenesis.
(TIF)

Figure S3 **Effect of tryptophan substitutions of FlhATM.** Motility assay of a flhA null mutant transformed with pUC19-based plasmids encoding various FlhA-substituted forms of FlhA in soft agar. Plates were incubated at 30°C for 6 hours. V, pUC19; WT, wild-type FlhA; D45W, FlhA(D45W); R85W, FlhA(R85W); R94W, FlhA(R94W); K203W, FlhA(K203W); R206W, FlhA(R206W); D208W, FlhA(D208W); D249W, FlhA(D249W); R270W, FlhA(R270W).
(TIF)

Figure S4 **Multiple sequence alignment of FliR homologs.** Conserved residues are labeled with various colors. Putative transmembrane helices were encircled by green boxes. UniProt Accession numbers: Salmonella (P54702); Escherichia (P33135); Yersinia (Q7CHY8); Pseudomonas (Q48GF7); Aquifex (O67773); Caulbacter (Q45975); Vibrio (Q5E3R1); Bacillus (P35537); Helicobacter (B5Z9U6); SpaR_Salmonella (P40701); YscT_Yersinia (P69984); SsaT_Salmonella (P96068). Stars indicate the positions of suppressor mutations.
(TIF)

Figure S5 **Characterization of flhA(K203W) suppression mutants.** (A) Allele specificity of the extragenic flhA(K203W) suppressor fliR alleles. Complementation test was carried out by P22-mediated transduction using a ΔfliH-fliI flhA(K203W) flhB(P28T) fliR::Tn10, ΔfliH-fliI flhA(K203A) flhB(P28T) fliR::Tn10 or ΔfliH-fliI flhA(R270W) flhB(P28T) fliR::Tn10 mutant strain as a recipient and a ΔfliH-fliI flhA(K203W) flhB(P28T) fliR(G103C) strain as a donor. Plates were incubated at 30°C for 40 hours. (B) Motility assay of SJW1103 (WT), NH0010 (fliR(G103C)), NH0011 (fliR(G103A)) and NH0012 (fliR(G117D)) in soft agar.
(TIF)

## References

[1] Macnab RM (2003). How bacteria assemble flagella.. Annu Rev Microbiol.

[2] Minamino T, Namba K (2004). Self-assembly and type III protein export of the bacterial flagellum.. J Mol Microbiol Biotechnol.

[3] Minamino T, Imada K, Namba K (2008). Mechanisms of type III protein export for bacterial flagellar assembly.. Mol Biosyst.

[4] Minamino T, Imada K, Namba K (2008). Molecular motors of the bacterial flagella.. Curr Opin Struct Biol.

[5] Hueck CJ (1998). Type III protein secretion systems in bacterial pathogens of animals and plants.. Microbiol Mol Biol Rev.

[6] Imada K, Minamino T, Tahara A, Namba K (2007). Structural similarity between the flagellar type III ATPase FliI and F1-ATPase subunits.. Proc Natl Acad Sci U S A.

[7] Ibuki T, Imada K, Minamino T, Kato T, Miyata T Common architecture between the flagellar protein export apparatus and F- and V-ATPases.. Nat Struct Mol Biol.

[8] Pallen MJ, Bailey CM, Beatson SA (2006). Evolutionary links between FliH/YscL-like proteins from bacterial type III secretion systems and second-stalk components of the FoF1 and vacuolar ATPases.. Protein Sci.

[9] Minamino T, Macnab RM (1999). Components of the Salmonella flagellar export apparatus and classification of export substrates.. J Bacteriol.

[10] Minamino T, Macnab RM (2000). Interactions among components of the Salmonella flagellar export apparatus and its substrates.. Mol Microbiol.

[11] Fan F, Ohnishi K, Francis NR, Macnab RM (1997). The FliP and FliR proteins of Salmonella typhimurium, putative components of the type III flagellar export apparatus, are located in the flagellar basal body.. Mol Microbiol.

[12] Kihara M, Minamino T, Yamaguchi S, Macnab RM (2001). Inter- genic suppression between the flagellar MS ring protein FliF of Salmonella and FlhA, a membrane component of its export apparatus.. J Bacteriol.

[13] Van Arnam JS, McMurry JL, Kihara M, Macnab RM (2004). Analysis of an engineered Salmonella flagellar fusion protein, FliR-FlhB.. J Bacteriol.

[14] Fan F, Macnab RM (1996). Enzymatic characterization of FliI. An ATPase involved in flagellar assembly in Salmonella typhimurium.. J Biol Chem.

[15] Minamino T, Macnab RM (2000). FliH, a soluble component of the type III flagellar export apparatus of Salmonella, forms a complex with FliI and inhibits its ATPase activity.. Mol Microbiol.

[16] González-Pedrajo B, Fraser GM, Minamino T, Macnab RM (2002). Molecular dissection of Salmonella FliH, a regulator of the ATPase FliI and the type III flagellar protein export pathway.. Mol Microbiol.

[17] Thomas J, Stafford GP, Hughes C (2004). Docking of cytosolic chaperone-substrate complexes at the membrane ATPase during flagellar type III protein export.. Proc Natl Acad Sci U S A.

[18] Evans LDB, Stafford GP, Ahmed S, Fraser GM, Hughes C (2006). An escort mechanism for cycling of export chaperones during flagellum assembly.. Proc Natl Acad Sci U S A.

[19] Imada K, Minamino T, Kinoshita M, Furukawa Y, Namba K (2010). Structural insight into the regulatory mechanisms of interactions of the flagellar type III chaperone FliT with its binding partners.. Proc Natl Acad Sci U S A.

[20] Minamino T, Gonzalez-Pedrajo B, Kihara M, Namba K, Macnab RM (2003). The ATPase FliI can interact with the type III flagellar protein export apparatus in the absence of its regulator, FliH.. J Bacteriol.

[21] Minamino T, Yoshimura SD, Morimoto YV, González-Pedrajo B, Kami-ike N (2009). Roles of the extreme N-terminal region of FliH for efficient localization of the FliH-FliI complex to the bacterial flagellar type III export apparatus.. Mol Microbiol.

[22] Minamino T, Namba K (2008). Distinct roles of the FliI ATPase and proton motive force in bacterial flagellar protein export.. Nature.

[23] Minamino T, Shimada M, Okabe M, Saijo-Hamano Y, Imada K (2010). Role of the C-terminal cytoplasmic domain of FlhA in bacterial flagellar type III protein export.. J Bacteriol.

[24] Paul K, Erhardt M, Hirano T, Blair DF, Hughes KT (2008). Energy source of flagellar type III secretion.. Nature.

[25] Barker CS, Meshcheryakova IV, Kostyukova AS, Samatey FA (2010). FliO regulation of FliP in the formation of the Salmonella enterica flagellum.. PLoS Genet.

[26] Minamino T, Iino T, Kutuskake K (1994). Molecular characterization of the Salmonella typhimurium flhB operon and its protein products.. J Bacteriol.

[27] McMurry JL, Arnam JSV, Kihara M, Macnab RM (2004). Analysis of the cytoplasmic domains of Salmonella FlhA and interactions with components of the flagellar export machinery.. J Bacteriol.

[28] Zhu K, Gonzalez-Pedrajo B, Macnab RM (2002). Interactions among membrane and soluble components of the flagellar export apparatus of Salmonella.. Biochemistry.

[29] Bange G, Kümmerer N, Engel C, Bozkurt G, Wild K (2010). FlhA provides the adaptor for coordinated delivery of late flagella building blocks to the type III secretion system.. Proc Natl Acad Sci U S A.

[30] Moore SA, Jia Y (2010). Structure of the cytoplasmic domain of the flagellar secretion apparatus component FlhA from Helicobacter pylori.. J Biol Chem.

[31] Saijo-Hamano Y, Imada K, Minamino T, Kihara M, Shimada M (2010). Structure of the cytoplasmic domain of FlhA and implication for flagellar type III protein export.. Mol Microbiol.

[32] Hirano T, Mizuno S, Aizawa S, Hughes KT (2009). Mutations in flk, flgG, flhA, and flhE that affect the flagellar type III secretion specificity switch in Salmonella enterica.. J Bacteriol.

[33] von Ballmoos C, Cook GM, Dimroth P (2008). Unique rotary ATP synthase and its biological diversity.. Annu Rev Biophys.

[34] Karlinsey JE, Tanaka S, Bettenworth V, Yamaguchi S, Boos W (2000). Completion of the hook-basal body complex of the Salmonella typhimurium flagellum is coupled to FlgM secretion and fliC transcription.. Mol Microbiol.

[35] Choe S, Stevens CF, Sullivan JM (1995). Three distinct structural environments of a transmembrane domain in the inwardly rectifying potassium channel ROMK1 defined by perturbation.. Proc Natl Acad Sci U S A.

[36] Sharp LL, Zhou J, Blair DF (1995). Features of MotA proton channel structure revealed by tryptophan-scanning mutagenesis.. Proc Natl Acad Sci U S A.

[37] Sharp LL, Zhou J, Blair DF (1995). Tryptophan-scanning mutagenesis of MotB, an integral membrane protein essential for flagellar rotation in Escherichia coli.. Biochem.

[38] Hoppe J, Schairer HU, Sebald W (1980). The proteolipid of a mutant ATPase from Escherichia coli defective in H+-conduction contains a glycine instead of the carbodiimide-reactive aspartyl residue.. FEBS Lett.

[39] Miller MJ, Oldenburg M, Fillingame H (1990). R.H. The essential carboxyl group in subunit c of the F1F0 ATP synthase can be moved and H+-translocating function retained.. Proc Natl Acad Sci U S A.

[40] Mitome N, Ono S, Sato H, Suzuki T, Sone N (2010). Essential arginine residue of the FO-a subunit in FOF1-ATP synthase has a role to prevent the proton shortcut without c-ring rotation in the FO proton channel.. Biochem J.

[41] Maloy SR, Nunn WD (1981). Selection for loss of tetracycline resistance by Escherichia coli.. J Bacteriol.

[42] Yamaguchi S, Fujita H, Sugata K, Taira T, Iino T (1984). Genetic analysis of H2, the structural gene for phase-2 flagellin in Salmonella.. J Gen Microbiol.

[43] Datsenko KA, Wanner BL (2000). One-step inactivation of chromosomal genes in Escherichia coli K-12 using PCR products.. Proc Natl Acad Sci U S A.

[44] Karlinsey JE, Hughes KT (2006). Genetic transplantation: Salmonella enterica Serovar Typhimurium as a host to study sigma factor and anti-sigma factor interactions in genetically intractable systems.. J Bacteriol.

[45] Saijo-Hamano Y, Minamino T, Macnab RM, Namba K (2004). Structural and functional analysis of the C-terminal cytoplasmic domain of FlhA, an integral membrane component of the type III flagellar protein export apparatus in Salmonella.. J Mol Biol.

