# Supplementary figures and images for: Genetic Characterization of Conserved Charged Residues in the Bacterial Flagellar Type III Export Protein FlhA
Source: PLoS One. 2011 Jul 19;6(7):e22417. doi: 10.1371/journal.pone.0022417 (PMC3139655; doi:10.1371/journal.pone.0022417)

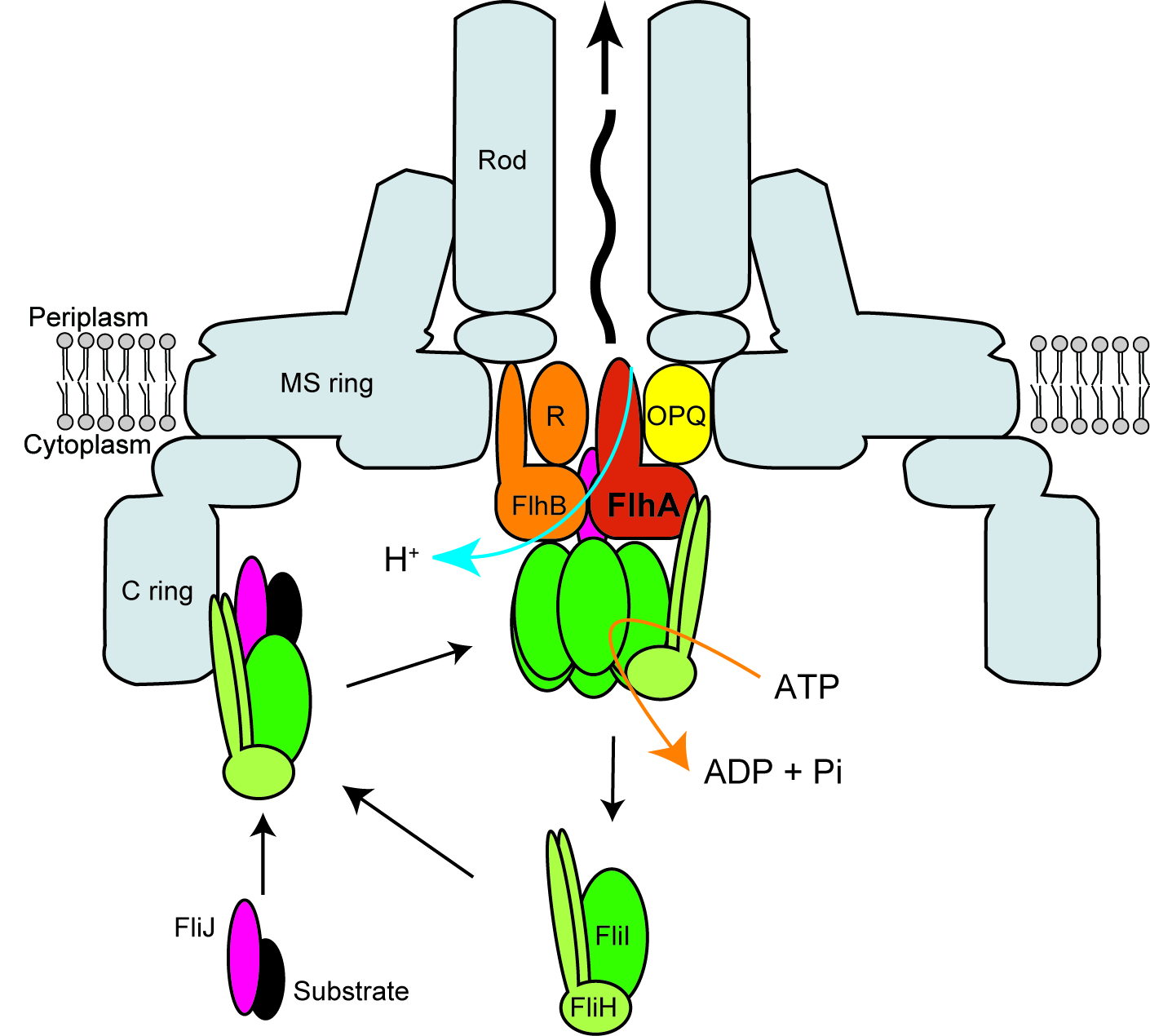

Supplement: Figure S1 — Schematic diagram of the bacterial flagellar type III protein export apparatus. The flagellar type III protein export apparatus consists of three soluble proteins, FliH, FliI, and FliJ and six integral membrane proteins, FlhA, FlhB, FliO, FliP, FliQ, and FliR. The integral membrane proteins are postulated to be located within the central pore of the MS ring and form the PMF-driven export gate complex. FlhA and FlhB have large cytoplasmic domains which project into the cavity within the C ring and form the docking platform for FliH, FliI, and FliJ as well as the substrates. FliI forms a heterotrimer with the FliH dimer in the cytoplasm. The FliH2FliI complex, along with FliJ and export substrates, is localized to the basal body C-ring through a specific interaction between FliH and FliN. FliI hexamerizes upon docking of the FliH-FliI-FliJ-substrate complex to the FlhA-FlhB platform and facilitates the entry of the N-terminal segment of a substrate into the gate. ATP hydrolysis by the FliI hexamer induces the dissociation of the FliHX-FliI6-FliJ complex from the gate. The export gate utilizes PMF across the cytoplasmic membrane as the energy source for the translocation of the export substrates into the central channel of the growing flagellar structure. (TIF) [file pone.0022417.s001.tif]

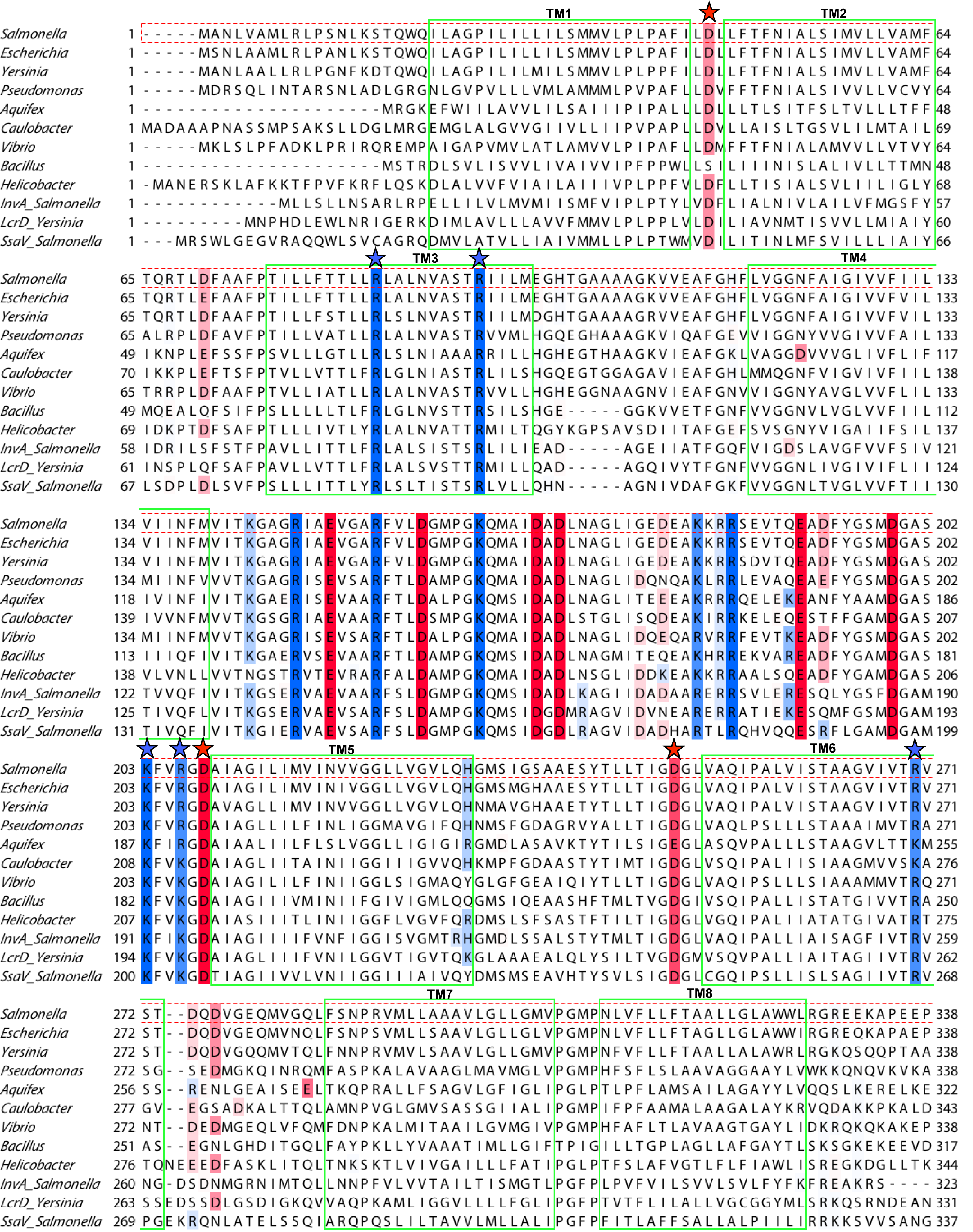

Supplement: Figure S2 — Multiple sequence alignment of FlhA homologs. Multiple sequence alignment was carried out by CLUSTAL-W (http://clustalw.ddbj.nig.ac.jp/top-j.html). Green boxes encircle putative transmembrane domains. UniProt Accession numbers: Salmonella (P40729); Escherichia (P76298); Yersinia (O56887); Pseudomonas (Q4KG43); Aquifex (O67265); Caulbacter (Q03845); Vibrio (Q9Z6F4); Bacillus (Q03845); Helicobacter (O06758); InvA_Salmonella (P0A1I3); LcrD_Yersinia (P66655); SsaV_Salmonella (P74856). Red and blue shades stars indicate conserved acidic and basic residues, respectively, which are selected for site-directed mutagenesis. (TIF) [file pone.0022417.s002.tif]

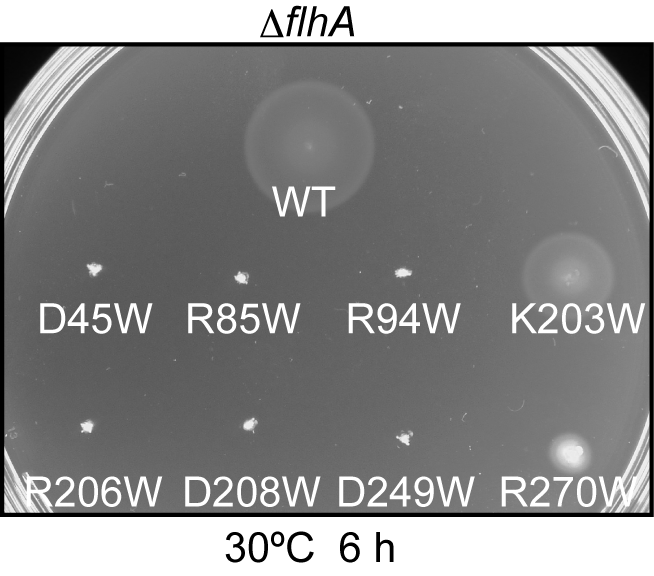

Supplement: Figure S3 — Effect of tryptophan substitutions of FlhATM. Motility assay of a flhA null mutant transformed with pUC19-based plasmids encoding various FlhA-substituted forms of FlhA in soft agar. Plates were incubated at 30°C for 6 hours. V, pUC19; WT, wild-type FlhA; D45W, FlhA(D45W); R85W, FlhA(R85W); R94W, FlhA(R94W); K203W, FlhA(K203W); R206W, FlhA(R206W); D208W, FlhA(D208W); D249W, FlhA(D249W); R270W, FlhA(R270W). (TIF) [file pone.0022417.s003.tif]

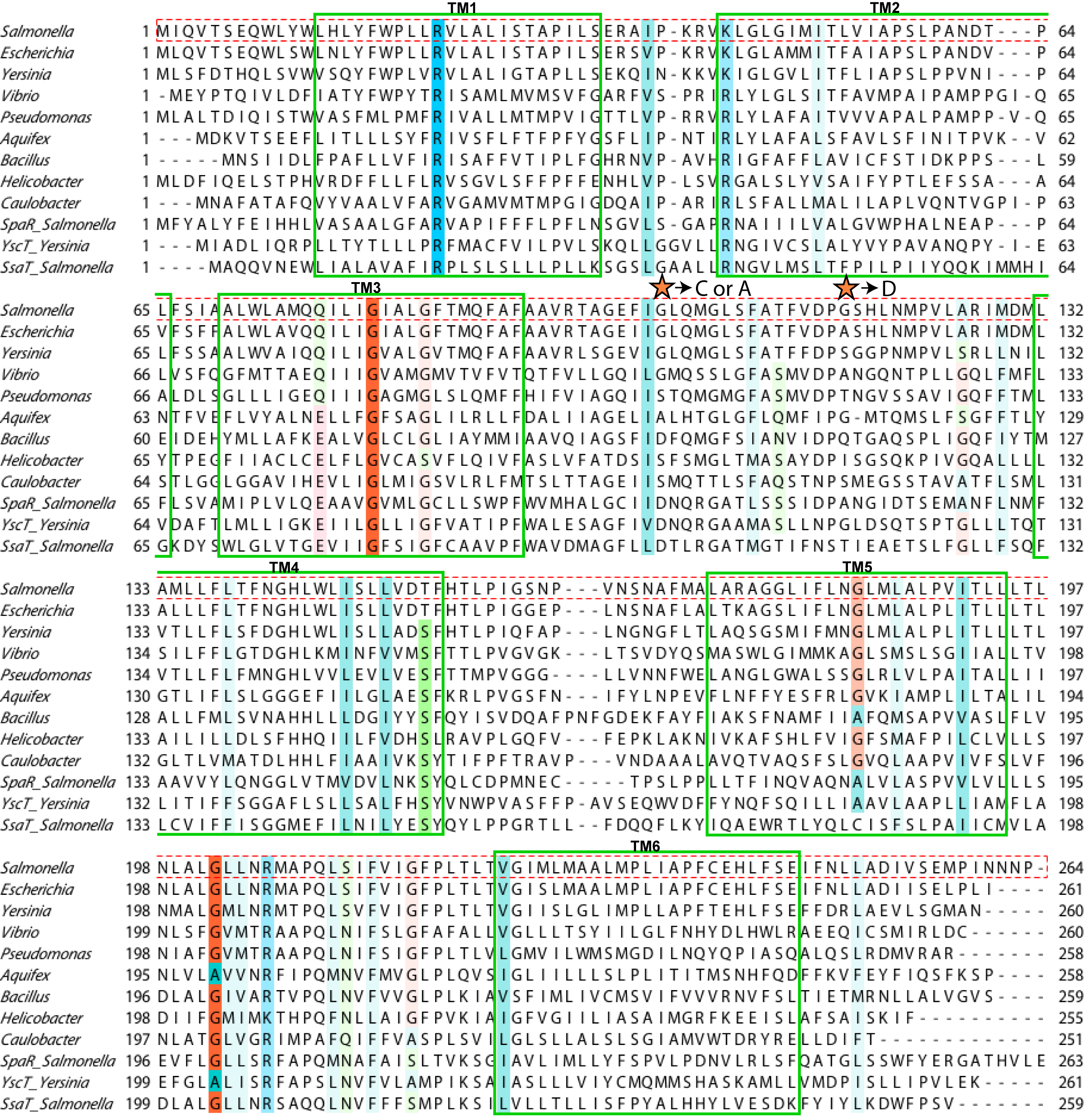

Supplement: Figure S4 — Multiple sequence alignment of FliR homologs. Conserved residues are labeled with various colors. Putative transmembrane helices were encircled by green boxes. UniProt Accession numbers: Salmonella (P54702); Escherichia (P33135); Yersinia (Q7CHY8); Pseudomonas (Q48GF7); Aquifex (O67773); Caulbacter (Q45975); Vibrio (Q5E3R1); Bacillus (P35537); Helicobacter (B5Z9U6); SpaR_Salmonella (P40701); YscT_Yersinia (P69984); SsaT_Salmonella (P96068). Stars indicate the positions of suppressor mutations. (TIF) [file pone.0022417.s004.tif]

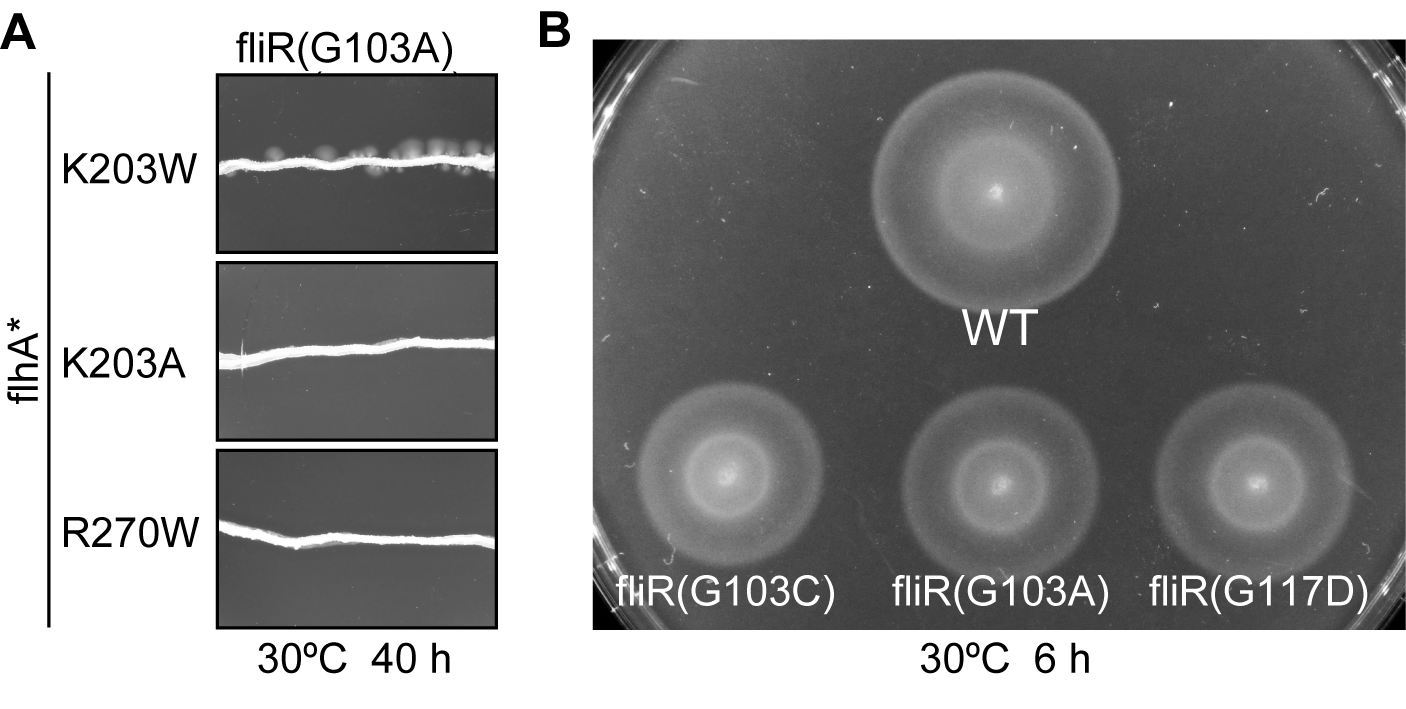

Supplement: Figure S5 — Characterization of flhA(K203W) suppression mutants. (A) Allele specificity of the extragenic flhA(K203W) suppressor fliR alleles. Complementation test was carried out by P22-mediated transduction using a ΔfliH-fliI flhA(K203W) flhB(P28T) fliR::Tn10, ΔfliH-fliI flhA(K203A) flhB(P28T) fliR::Tn10 or ΔfliH-fliI flhA(R270W) flhB(P28T) fliR::Tn10 mutant strain as a recipient and a ΔfliH-fliI flhA(K203W) flhB(P28T) fliR(G103C) strain as a donor. Plates were incubated at 30°C for 40 hours. (B) Motility assay of SJW1103 (WT), NH0010 (fliR(G103C)), NH0011 (fliR(G103A)) and NH0012 (fliR(G117D)) in soft agar. (TIF) [file pone.0022417.s005.tif]
